# Differential expression of alpha 2 macroglobulin in response to dietylstilbestrol and in ovarian carcinomas in chickens

**DOI:** 10.1186/1477-7827-9-137

**Published:** 2011-10-07

**Authors:** Whasun Lim, Wooyoung Jeong, Ji-Hye Kim, Jin-Young Lee, Jinyoung Kim, Fuller W Bazer, Jae Yong Han, Gwonhwa Song

**Affiliations:** 1 WCU Biomodulation Major, Department of Agricultural Biotechnology, Seoul National University, 599 Gwanak-ro, Gwanak-gu, Seoul 151-921, Korea; 2 Center for Animal Biotechnology and Genomics and Department of Animal Science, Texas A&M University, College Station, Texas 77843-2471, USA

**Keywords:** chicken, A2M, DES, cancer, oviduct, ovary

## Abstract

**Background:**

Alpha 2 macroglobulin (A2M; also known as ovostatin), a homotetrameric protein with four disulfide-linked subunits, has the unique feature of inactivating/inhibiting most known proteases including serine-, threonine-, cysteine-, aspartic- and metalloproteases. In chickens, A2M has been identified and characterized biochemically, but little is known of its functional role(s) in the oviduct, hormonal regulation of expression or its expression in ovarian carcinomas in chickens. Therefore, we investigated estrogen regulation of *A2M *gene expression during development of the chicken oviduct, and its expression in normal and cancerous ovaries from chickens.

**Methods:**

To determine tissue-specific expression of A2M in chickens, we collected various organs from male and female chickens and performed RT-PCR analyses. To examine A2M gene expression in the oviduct of 1-week-old female chicks that received a subcutaneous implant of 15 mg DES in the abdominal region for 20 days, we performed RT-PCR, qPCR and *in situ *hybridization analyses using cDNAs from control- (n = 5) and DES-treated oviducts (n = 5), and then each segment of the oviduct from DES-treated chicks. To determine if A2M is a biomarker of ovarian cancer in hens, we collected cancerous (n = 10) ovaries from a total of 136 chickens which had completely stopped egg-laying and performed RT-PCR and *in situ *hybridization analyses.

**Results:**

We found that *A2M *is most abundant in the chicken oviduct, specifically luminal (LE) and glandular epithelia (GE), but it was not detected in any other tissues of either sex. We then determined that DES (dietylstilbestrol, a synthetic nonsteroidal estrogen) increased *A2M *mRNA only in LE and GE of the oviduct of chicks. Further, expression of *A2M *was most abundant in GE of endometrioid adenocarcinoma of cancerous, but not normal ovaries of hens.

**Conclusions:**

Collectively, results of the present study indicate that *A2M *is novel estrogen-stimulated gene expressed in LE and GE of the chicken oviduct and may be used for monitoring effects of therapies for ovarian cancer in laying hens.

## Background

The alpha 2 macroglobulins (A2M) are proteins in blood that act as protease inhibitors in mammals [1-4]. In humans, the *A2M *gene is a single-copy gene located on chromosome 12p12-13 that encodes for a functional homotetramer protein with disulfide-linked 180-kDa subunits [5,6]. Even though A2M is produced predominantly by the liver, it may also be expressed in the reproductive tract, heart, and brain, and may have important roles in many physiological processes and medical illnesses including Alzheimer's disease [4,7,8]. Of particular note, A2M increase in blood serum of women with inflammatory and neoplastic lesions of the ovary [9]. It also stimulates production of activin and inhibin in pre-ovulatory follicles [10,11] and controls coordinate changes in uterine vasculature during pregnancy [4]. In addition, Umans *et al*. reported that *A2m*-deficient mice were viable and produced normal size litters with normal sex ratios over three generations [8]. Moreover, A2M regulates the function of cortical granule proteases and other trypsin-like proteases activated in sea urchin eggs during fertilization [12]. In chickens, A2M is also known as an ovostatin or ovomacroglobulin. It is found in the oviduct and egg white, but not in other tissues or serum [13] and it has a strong anti-collagenase activity [14,15]. However, little is known about regulation of its expression by steroid hormones in the oviduct or its expression in normal and cancerous ovaries.

As the primary female sex hormone, estrogen regulates reproductive behavior. It is responsible for proliferation and differentiation of several cell types associated with osteoporosis, diabetes, cardiovascular disease, and reproductive carcinomas [16-21]. The chicken oviduct is well-known as an excellent research model for studies of organ development and hormonal responsiveness [16]. During development of the chicken oviduct, estrogen stimulates proliferation and cytodifferentiation of epithelial cells to tubular gland cells and expression of oviduct-specific genes [22,23]. However, progesterone interferes with normal estrogen-mediated cytodifferentiation of tubular gland cells [24-27]. Estrogen also affects calcium metabolism for eggshell formation and ovipositioning or egg laying [28,29]. In addition, estrogen administration to sexually immature chicks stimulates growth of the oviduct by inducing cellular hyperplasia and hypertrophy [16,30]. The mammalian oviduct undergoes diverse biological changes in response to sex steroids during the estrous cycle and early pregnancy as these actions are pivotal to establishing an optimal microenvironment for events ranging from gamete transport to early embryonic development [31]. To investigate the biological actions and signaling pathways of estrogen, the chicken is one of the best animal models [16]. Indeed, steroid hormones are involved in many physiological and developmental processes accompanying modification of tissue-specific and conditional control of gene expression and homeostasis [16,32]. Although general effects of estrogen and progesterone on the reproductive tract of vertebrates are well documented, details of their interactions that affect cell signaling pathways in avian species are unclear.

The objectives of this study were to: 1) determine tissue- and cell-specific expression of the *A2M *gene in chickens; 2) determine whether estrogen regulates expression of *A2M *during oviductal development in chicks; and 3) compare expression of *A2M *in normal and cancerous ovaries from laying hens. Results of this study indicate that *A2M *is a novel estrogen-stimulated gene during development of the chicken oviduct and that it may be an initial candidate gene for further study of the development of epithelial ovarian cancer in hens.

## Methods

### Experimental animals and animal care and use

The experimental use of chickens for this study was approved by the Institute of Laboratory Animal Resources, Seoul National University (SNU-070823-5). White Leghorn (WL) chickens were subjected to standard management practices at the University Animal Farm, Seoul National University, Korea with respect to management of hens for reproduction, incubation of eggs and rearing of chicks, as well as standard operating protocols in our laboratory. All chickens had *ad libitum *access to feed and water.

### Tissue samples

#### Study one

Following euthanasia of mature WL hens, tissue samples were collected from brain, heart, liver, kidney, muscle, small intestine, gizzard, ovary, oviduct and testis of 1- to 2-year-old males (n = 3) and females (n = 3). Subsets of these samples were frozen or fixed in 4% paraformaldehyde for further analyses. Frozen tissue samples were cut into 5- to 7-mm pieces, frozen in liquid nitrogen vapor, and stored at -80°C. The other samples were cut into 10 mm pieces and fixed in fresh 4% paraformaldehyde in PBS (pH 7.4). After 24 h, fixed tissues were changed to 70% ethanol for 24 h and then dehydrated and embedded in Paraplast-Plus (Leica Microsystems, Wetzlar, Germany). Paraffin-embedded tissues were sectioned at 5 μm.

#### Study two

Female chicks were identified by PCR analysis using W chromosome-specific primer sets [33]. Treatment with DES and recovery of the oviduct were conducted as reported previously [34,35]. Briefly, a 15 mg DES pellet was implanted subcutaneously in the abdominal region of 1-week-old female chicks for release of hormone for 20 days [34,36,37]. Five chicks in each group were euthanized using 60%-70% carbon dioxide. Subsets of these samples were frozen or fixed in 4% paraformaldehyde for further analyses. Frozen tissue samples were cut into 5- to 7-mm pieces and frozen in liquid nitrogen. The other samples were cut into 10- to 15-mm pieces and fixed in fresh 4% paraformaldehyde in PBS (pH 7.4). After 24 h, fixed tissues were changed to 70% ethanol for 24 h and then dehydrated and embedded in Paraplast-Plus (Leica Microsystems, Wetzlar, Germany). Paraffin-embedded tissues were sectioned at 5 μm.

#### Study three

In this study, a total of 136 chickens (88 chickens over 36 months of age and 48 chickens over 24 months of age), which had completely stopped laying eggs were euthanized for biopsy and cancerous (n = 10) ovaries were collected. As a control, normal (n = 5) ovaries were also collected from egg-laying hens. We examined tumor stage in 10 hens with cancerous ovaries based on characteristic features of chicken ovarian cancers [38]. In three hens, ovarian tumor cells were classified as Stage III as they had metastasized to the gastrointestinal tract and superficial surface of the liver with profuse ascites in the abdominal cavity. In five hens, the tumors had metastasized to distant organs such as liver parenchyma, lung, gastrointestinal tract and oviduct with profuse ascites, so these were classified at Stage IV tumors. The other two hens did not have tumors in any other organs; therefore, their ovarian tumors were classified as Stage I. Subsets of these samples were frozen or fixed in 4% paraformaldehyde for further analyses. Frozen tissue samples were cut into 5- to 7-mm pieces and frozen in liquid nitrogen. The other samples were cut into 10 mm pieces and fixed in 4% paraformaldehyde in PBS (pH 7.4). After 24 h, fixed tissues were changed to 70% ethanol for 24 h and then dehydrated and embedded in Paraplast-Plus (Leica Microsystems, Wetzlar, Germany). Paraffin-embedded tissues were sectioned at 5 μm and stained with hematoxylin and eosin. Epithelial ovarian cancers in chickens were classified based on their cellular subtypes and patterns of cellular differentiation with reference to ovarian malignant tumor types in humans [38].

### RNA isolation

Total cellular RNA was isolated from frozen tissues using Trizol reagent (Invitrogen, Carlsbad, CA) according to manufacturer's recommendations. The quantity and quality of total RNA was determined by spectrometry and denaturing agarose gel electrophoresis, respectively.

### Sequence analysis

For pair-wise comparisons, the amino acid sequences of *A2M *genes from each species were aligned using Geneious Pro Version 5.04 [39] with default penalties for gap and the protein weight matrix of BLOSUM (Blocks Substitution Matrix).

### Semiquantitative RT-PCR analysis

The level of expression of *A2M *mRNA in various organs from chickens, including the oviduct, was assessed using semi-quantitative RT-PCR as described previously [40]. The cDNA was synthesized from total cellular RNA (2 ug) using random hexamer (Invitrogen, Carlsbad, CA) and oligo (dT) primers and AccuPower^® ^RT PreMix (Bioneer, Daejeon, Korea). The cDNA was diluted (1:10) in sterile water before use in PCR. For *A2M*, the sense primer (5'-CTG GCT CAC TGC CTT TGT GT-3') and antisense primer (5'-CCG TCA ACT TCC TTT GCT GA-3') amplified a 405-bp product. For *GAPDH *(housekeeping gene; glyceraldehyde 3-phosphate dehydrogenase), the sense primer (5'-TGC CAA CCC CCA ATG TCT CTG TTG-3') and antisense primer (5'-TCC TTG GAT GCC ATG TGG ACC AT-3') amplified a 301-bp product. The primers, PCR amplification and verification of their sequences were conducted as described previously [40]. PCR amplification was conducted using approximately 60 ng cDNA as follows: (1) 95°C for 3 min; (2) 95°C for 20 sec, 61°C for 40 sec, and 72°C for 1 min for 35 cycles (*A2M*), 30 cycles (*GAPDH*); and (3) 72°C for 5 min. Then, equal amounts of reaction product were analyzed using a 1% agarose gel, and PCR products were visualized using ethidium bromide staining. The amount of DNA present was quantified by measuring the intensity of light emitted from correctly sized bands under ultraviolet light using a Gel Doc™ XR+ system with Image Lab™ software (Bio-Rad).

### Quantitative RT-PCR analysis

Total RNA was extracted from each oviduct of control and DES-treated chicks using TRIzol (Invitrogen) and purified using an RNeasy Mini Kit (Qiagen). Complementary DNA was synthesized using AccuPower^® ^RT PreMix (Bioneer, Daejeon, Korea). Gene expression levels were measured using SYBR^® ^Green (Sigma, St. Louis, MO, USA) and a StepOnePlus™ Real-Time PCR System (Applied Biosystems, Foster City, CA, USA). The *GAPDH *gene was simultaneously analyzed as a control and used for normalization of data. Expression of each target gene and *GAPDH *was analyzed in triplicate. Using the standard curve method, we determined levels of expression of the examined genes using the standard curves and C_T _values, and normalized them based on *GAPDH *expression levels. The PCR conditions were 95°C for 3 min, followed by 40 cycles at 95°C for 20 sec, 60°C for 40 sec, and 72°C for 1 min using a melting curve program (increasing the temperature from 55°C to 95°C at 0.5°C per 10 sec) and continuous fluorescence measurement. The ROX dye (Invitrogen) was used as a negative control for measurements of fluorescence. Sequence-specific products were identified by generating a melting curve in which the C_T _value represented the cycle number at which a fluorescent signal was significantly greater than background, and relative gene expression was quantified using the 2^-ΔΔCT ^method [41]. For the control, the relative quantification of gene expression was normalized to the C_T _of the control oviduct.

### *In situ *hybridization analysis

For hybridization probes, PCR products were generated from cDNA with the primers used for RT-PCR analysis. The products were gel-extracted and cloned into pGEM-T vector (Promega). After verification of the sequences, plasmids containing gene sequences were amplified with T7- and SP6-specific primers (T7:5'-TGT AAT ACG ACT CAC TAT AGG G-3'; SP6:5'-CTA TTT AGG TGA CAC TAT AGA AT-3') then digoxigenin (DIG)-labeled RNA probes were transcribed using a DIG RNA labeling kit (Roche Applied Science, Indianapolis, IN). Tissues were collected and fixed in 4% paraformaldehyde. The tissues were embedded in paraffin and sectioned at 5 μm on APES-treated (silanized) slides. The sections were then deparaffinized in xylene and rehydrated to diethylpyrocarbonate (DEPC)-treated water through a graded series of alcohol. The sections were treated with 1% Triton X-100 in PBS for 20 min and washed two times in DEPC-treated PBS. After washing in DEPC-treated PBS, the sections were digested with 5 μg/ml Proteinase K (Sigma) in TE buffer (100 mM Tris-HCl, 50 mM EDTA, pH 8.0) at 37°C. After post-fixation in 4% paraformaldehyde, sections were incubated twice for 5 min each in DEPC-treated PBS and incubated in TEA buffer (0.1 M triethanolamine) containing 0.25% (v/v) acetic anhydride. The sections were incubated in a prehybridization mixture containing 50% formamide and 4× standard saline citrate (SSC) for at least 10 min at room temperature. After prehybridization, the sections were incubated with a hybridization mixture containing 40% formamide, 4× SSC, 10% dextran sulfate sodium salt, 10 mM DTT, 1 mg/ml yeast tRNA, 1 mg/ml salmon sperm DNA, 0.02% Ficoll, 0.02% polyvinylpyrrolidone, 0.2 mg/ml RNase-free bovine serum albumin and denatured DIG-labeled cRNA probe for overnight at 42°C in a humidified chamber. After hybridization, sections were washed for 15 min in 2× SSC at 37°C, 15 min in 1× SSC at 37°C, 30 min in NTE buffer (10 mM Tris, 500 mM NaCl and 1 mM EDTA) at 37°C and 30 min in 0.1× SSC at 37°C. After blocking with 2% normal sheep serum (Santa Cruz Biotechnology, INC.), the sections were incubated overnight with sheep anti-DIG antibody conjugated to alkaline phosphatase (Roche). The signal was visualized by exposure to a solution containing 0.4 mM 5-bromo-4-chloro-3-indolyl phosphate, 0.4 mM nitroblue tetrazolium, and 2 mM levamisole (Sigma).

### Statistical analyses

Differences in the variance between control and DES-treated oviducts were analyzed by analysis of variance, and differences between means were subjected to Student's *t *test. The probability value of *P *< 0.05 was considered statistically significant. Excel (Microsoft, Redmond, WA, USA) was used for statistical analyses.

## Results

### Multiple sequence alignment, pairwise comparisons, and phylogenetic analysis

The chicken *A2M *gene was found in the genomic region spanning 35,791 bp on chromosome 1. The gene consists of 35 exons and the mRNA has 4,715 bp encoding a protein with 1,454 amino acid residues. The primary sequence of chicken A2M was compared to those of some mammalian species. In pairwise comparisons of chicken A2M with eight other vertebrate species, chicken A2M protein has moderate homology to mammalian A2M proteins (42.4-45.4%, Table 1). In particular, chicken A2M protein contains the highly conserved macroglobulin 1 (MG1) and MG2 domains, A2M family N-terminal region, and A2M receptor binding domain found in mammalian A2M proteins (Additional file 1, Supplemental Figure S1). In the phylogenetic tree generated from primary sequences of available vertebrate A2M proteins, chicken A2M was placed between mammalian and amphibian species consistent with the general pattern of molecular evolution in vertebrates (Additional file 2, Supplemental Figure S2).

**Table 1:** Pairwise comparisons of A2M among chicken and several mammalian species

Species	Symbol	Identity (%)	**Accession NO**.
*Gallus gallus *(Chicken)	A2M	-	NP_990557.1
vs. *Homo sapiens *(Human)	A2M	45.2	NP_000005.2
vs. *Pan troglodytes *(Chimpanzee)	A2M	45.4	XP_001139819.1
vs. *Pongo abelii *(Orangutan)	A2M	45.2	NP_001126929.1
vs. *Macaca mulatta *(Rhesus monkey)	A2M	45.1	XP_001114328.1
vs. *Mus musculus *(Mouse)	a2m	43.2	NP_783327.2
vs. *Rattus norvegicus *(Rat)	A2M	43.5	NP_036620.2
vs. *Bos taurus *(Cow)	A2M	42.5	NP_001103265.1
vs. *Xenopus laevis *(Frog)	A2M	42.4	NP_001165531.1

### *A2M *mRNA expression in chickens (study one)

**Figure 1:**
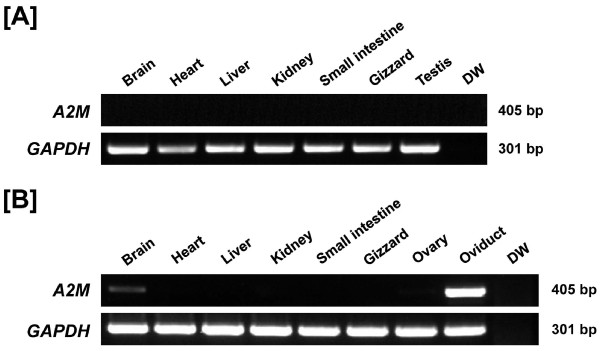
**Expression of A2M in various organs of male and female chickens**. Results of RT-PCR analysis using cDNA from different organs of male [A] and female [B] chickens indicate that *A2M *mRNA is expressed abundantly in the oviduct and, to a lesser extent, in the brain of female chickens. However, specific expression was not detected in any other organs analyzed for either sex.

To determine tissue-specific expression of *A2M *mRNA in chickens, we collected various organs from male and female chickens and performed RT-PCR analysis. As shown in Figure 1, *A2M *mRNA is abundantly expressed in the oviduct and, to a lesser extent, in the brain of female chickens. However, specific expression was not detected in any other organs analyzed for either sex. Therefore, further studies focused on A2M in the chicken oviduct.

### Localization of A2M mRNA expression in the chicken oviduct

**Figure 2:**
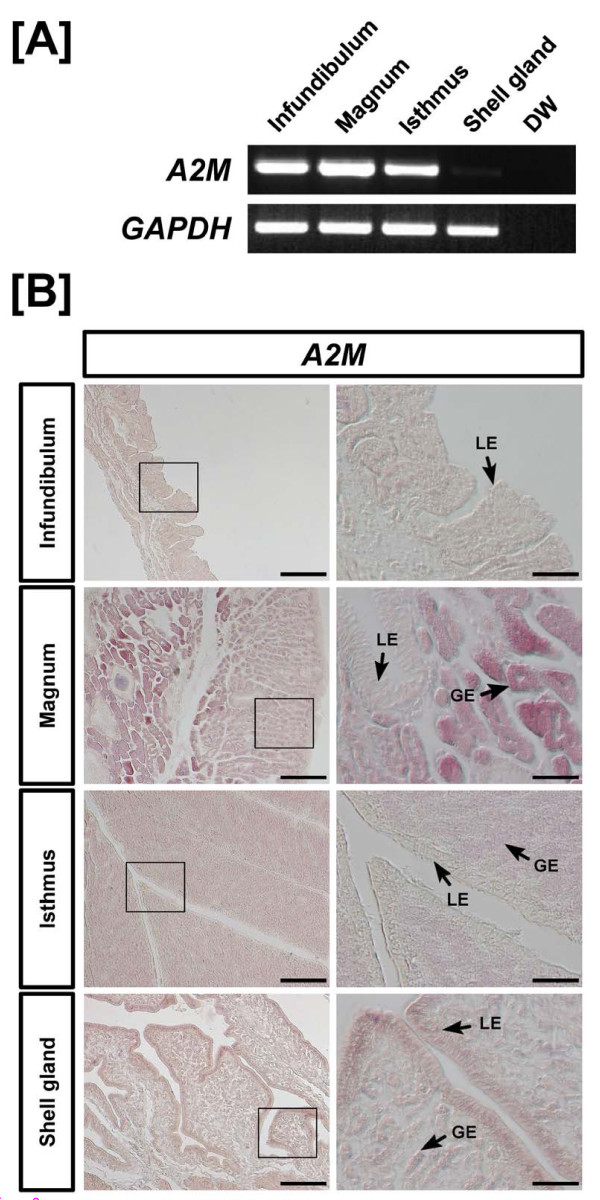
**RT-PCR and *in situ *hybridization analyses of *A2M *mRNAs in the chicken oviduct**. Results of RT-PCR analysis using cDNA from different segments from female chicken [A] indicate that *A2M *mRNA is expressed abundantly in the infundibulum, magnum, and isthmus of the oviduct. At lower abundance, *A2M *mRNA is expressed in shell gland. [B] Cross-sections of the infundibulum, magnum, isthmus and shell gland of the chicken oviduct hybridized with antisense or sense chicken *A2M *cRNA probes revealed that *A2M *mRNA is most abundant in the glandular epithelium (GE) of the magnum, and lesser abundant in GE of the isthmus and luminal epithelium (LE) and GE of the shell gland. Legend: LE, luminal epithelium; GE, glandular epithelium; *Scale bar *represents 200 μm (the first columnar panels) and 50 μm (the second columnar panels).

The oviduct of egg-laying hens includes the infundibulum (site of fertilization), magnum (production of components of egg-white), isthmus (formation of the soft shell membrane), and shell gland (formation of the egg shell). Using RT-PCR analysis, it was determined that *A2M *mRNA is expressed abundantly in the infundibulum, magnum, and isthmus of the chicken oviduct (Figure 2A). At a lower abundance, *A2M *mRNA is also expressed in the shell gland. In addition, *in situ *hybridization analysis revealed that *A2M *mRNA is most abundant in glandular epithelium (GE) of the magnum but expressed to a lesser extent in GE of the isthmus and luminal epithelium (LE) and GE of the shell gland (Figure 2B). Little or no mRNA was detected in stromal cells, blood vessels, immune cells or myometrium (smooth muscle) of the oviduct.

### Effects of DES on A2M mRNA expression in the chicken oviduct (study two)

**Figure 3:**
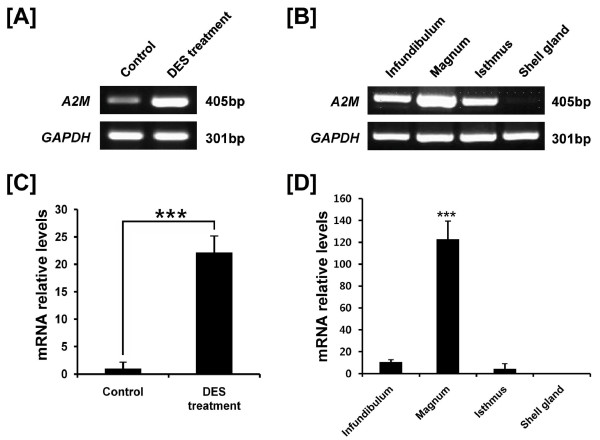
**Effect of DES on tissue specific expression of chicken *A2M *based on RT-PCR and qPCR analyses**. Both RT-PCR [A and B] and q-PCR [C and D] analyses were performed using cDNA templates from DES-treated and control chicken oviducts (mean ± SEM; P < 0.001) to determine that DES induced about a 22-fold increase in oviductal *A2M *mRNA as compared to control chicks.

**Figure 4:**
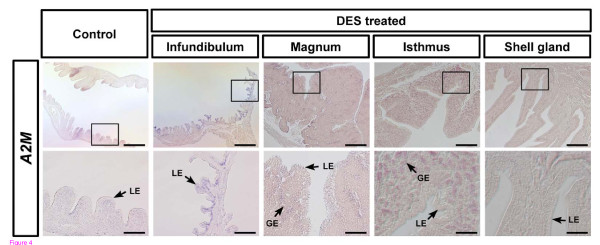
***In situ *hybridization analyses of *A2M *mRNA in oviducts of DES-treated and control chicks**. *In situ *hybridization with cross-sections of the infundibulum, magnum, isthmus, and shell gland of the chicken oviduct with antisense or sense chicken *A2M *cRNA probes revealed abundant expression of *A2M *mRNA only in glandular epithelium (GE) of isthmus and lower expression in GE of the magnum of chick oviducts treated with DES. Legend: LE, luminal epithelium; GE, glandular epithelium; *Scale bar *represents 200 μm (the first horizontal panels) and 50 μm (the second horizontal panels).

Oviduct-specific and cell-type specific expression of *A2M *in the oviductal segments of hens suggested regulation by estrogen during development of the oviduct in chicks. We reported that DES induces development and differentiation of the chicken oviduct [35] and discovered candidate genes regulating oviduct development in chickens [30]. Therefore, we examined *A2M *gene expression in the oviduct of 37-day-old chicks that received a subcutaneous implant of 15 mg DES in the abdominal region for 20 days [35]. We performed RT-PCR analyses using cDNAs from control- (n = 5) and DES-treated oviducts (n = 5), and then each segment of oviducts from DES-treated chicks (Figure 3A and -3B). In addition, as illustrated in Figure 3C, qPCR analysis revealed that DES stimulated a 22-fold increase (P < 0.001) in *A2M *mRNA in oviducts as compared to oviducts from control chicks. DES treatment also stimulated a 123-fold increase (P < 0.001) in *A2M *mRNA in the magnum (Figure 3D). Further, *in situ *hybridization analyses revealed abundant expression of *A2M *mRNA only in GE of the isthmus and a lower abundance in GE of the magnum of chick oviducts treated with DES (Figure 4).

### Differential expression of A2M mRNA in normal and cancerous ovaries of hens (study three)

**Figure 5:**
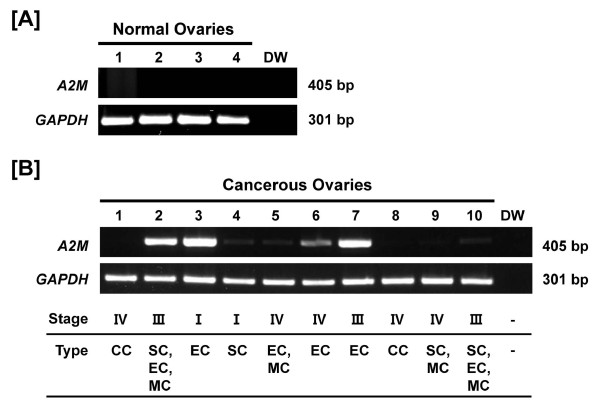
**Expression and quantitation of *A2M *mRNA in normal and cancerous ovaries from hens**. [A] RT-PCR analyses were performed using cDNA templates from normal and cancerous ovaries of chickens with chicken *A2M *and *GAPDH*-specific primers. Lanes 1 to 4 show results of analysis of four normal ovaries with distilled water as a negative control. [B] Lanes 1-10 are from analyses of 10 different cancerous ovaries. Expression of *A2M *mRNA was predominantly in endometrioid carcinoma, with little or no expression in serous, mucinous or clear cell carcinomas and normal ovaries (mean ± SEM; P < 0.05). Legend for panel B: Lane 1, clear cell carcinoma (Stage IV); Lane 2, endometrioid/serous/mucinous carcinoma (Stage III); Lane 3, endometrioid carcinoma (Stage I); Lane 4, serous carcinoma (Stage I); Lane 5, mucinous/endometrioid carcinoma (Stage IV); Lane 6, endometrioid carcinoma (Stage IV); Lane 7, endometrioid carcinoma (Stage III); Lane 8, clear cell carcinoma (Stage IV); Lane 9, serous/mucinous carcinoma (Stage IV); and Lane 10, serous/mucinous/endometrioid carcinoma (Stage III).

**Figure 6:**
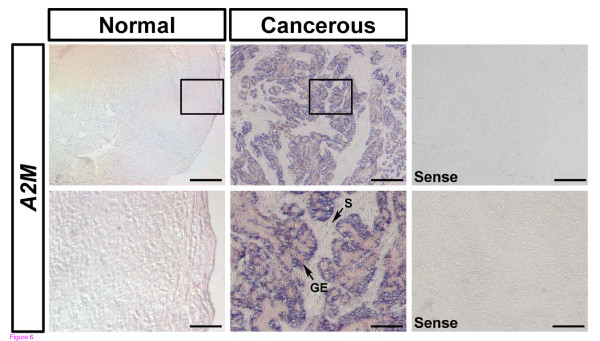
***In situ *hybridization analyses of *A2M *mRNA in normal and cancerous ovaries of hens**. Cross-sections of normal and cancerous ovaries of hens hybridized with antisense or sense chicken *A2M *cRNA probes demonstrated abundant *A2M *mRNA predominantly in GE of cancerous ovaries, but not in LE, stroma or blood vessels. Legend: F, follicle; GE, glandular epithelium; S, stroma; *Scale bar *represents 200 μm (the first horizontal panels and sense) or 50 μm (the second horizontal panels and sense).

It was next determined if A2M is a biomarker of ovarian cancer in laying hens which is the only animal model that spontaneously develops epithelia-derived ovarian cancer at a high rate as occurs in women [42]. As illustrated in Figure 5B, RT-PCR analyses indicated that *A2M *mRNA expression was abundant in endometrioid carcinomas (lane 2, -3, -6, and -7), which is characterized by glandular patterns resembling those of the endometrium, but there was little or no expression of A2M in serous, mucinous or clear cell carcinomas and normal ovaries (please see Additional file 3, Supplemental Figure S3). Further, *in situ *hybridization analysis revealed abundant *A2M *mRNA localized predominantly to GE of cancerous ovaries, but not LE, stroma or blood vessels (Figure 6).

## Discussion

Results of the present study are the first to demonstrate tissue- and cell-specific expression of *A2M *mRNA in oviducts from normal chickens and to identify high levels of expression of *A2M *gene in cancerous ovaries of laying hens. Expression of *A2M *mRNA is unique to the chicken oviduct. In humans, A2M is synthesized primarily in the liver [43,44] and as a major plasma protein, it makes up roughly 8-10% of the total protein in human blood [45,46]. However, in chickens, A2M (also known as ovomacroglobulin or ovostatin) is synthesized in the oviduct and found in egg whites [14,47,48]. Indeed, the magnum segment of the chicken oviduct synthesizes and secretes components of egg-white such as ovalbumin, conalbumin, lysozyme, and ovomucoid [49] and the isthmus is the site of formation of the soft shell membrane. As illustrated in Figure 2, *A2M *mRNA was most abundant in GE of the magnum and isthmus, but also expressed to a lesser extent in both LE and GE of the shell gland. Given the high level of expression of *A2M *in these two segments of the oviduct, A2M may interact with other proteases to maintain a balance of proteases and protease inhibitors involved in egg formation after ovulation.

It is well known that estrogen is required for normal development of reproductive organs in female mammals and birds [36]. In general, the biological actions of estrogen are mediated by its cognate receptors, estrogen receptors alpha and beta which activate and recruit a variety of transcription factors that bind to estrogen response elements in the 5' upstream region of target genes [16,17]. Indeed, several steroid hormones, including estrogen, are involved in many physiological and developmental events requiring modification of cell-type and tissue-specific gene expression [16,32]. Consistent with these results, we reported that exogenous DES affects growth, development and differentiation of the chicken oviduct [35] and discovered candidate genes and pathways regulating oviduct development in chickens [30]. In this study, DES induced *A2M *mRNA in GE of the infundibulum, magnum, and isthmus of the chick oviduct. These results indicate that A2M is likely regulated by estrogen in the oviduct and that it is likely involved in growth, differentiation and function of the chicken oviduct.

Ovarian cancer is the most lethal gynecological malignancy and the leading cause of cancer-related deaths among woman [50-52], but it is rarely diagnosed at an early stage [53]. Therefore, over 75% of woman diagnosed are at an advanced stage of the disease because it is generally asymptomatic [54] and there is no specific biomarker(s) for early detection [38,55]. Although genetically manipulated rodent models have been used to elucidate some aspects of the etiologies and pathogenesis of ovarian cancer, the non-spontaneous feature of their ovarian cancer limits its clinical application [38,56,57]. Meanwhile, the laying hen is a unique animal model for research on human ovarian cancer, because laying hens spontaneously develop epithelial cell-derived ovarian cancer as occurs in women [38,42,58]. We found that *lysosomal cysteine cathepsin B *(*CTSB*) [59] and *serpin peptidase inhibitor, clade B, member 11 *(*SERPINB11*) (unpublished data) are highly expressed in cancerous ovaries of hens. In the present study, as for SERPINB11, A2M is predominantly localized in endometrioid carcinoma, but there was little or no expression in serous, mucinous or clear cell carcinomas and normal ovaries (Figure 5A and-5B). In addition, *A2M *mRNA is abundant in GE of cancerous ovaries, but not in LE, stroma or blood vessels. These results strongly support the idea that proteases and their inhibitors play fundamental roles in a wide variety of biological events including cancer progression and metastasis due to their capacity to degrade extracellular matrix proteins [60]. Although human A2M is a well known major cytotoxic factor in serum which inhibits the DNA synthesis in mouse ovarian tumor cells [61,62], Hawkridge and co-workers [63] reported that A2M increased in plasma over a 6 month period in laying hens with late-stage epithelial ovarian cancer. This result provides protein-level evidence for up-regulation of a predicted form of A2M in plasma of laying hens that developed Stage IV ovarian cancer as compared to concentrations in plasma of healthy control laying hens. Collectively, our current results indicate that A2M is likely involved in gland morphogenesis and angiogenesis in chicken carcinogenesis. Alternatively, it is possible that A2M interacts reciprocally with other proteases in cancerous ovaries of chickens as it exhibits some inhibitory activity against a variety of proteinases (including serine-, cysteine-, aspartic- and metalloproteinases) [64].

Results of the present study indicate that *A2M *is a novel estrogen-regulated gene during development of the chicken oviduct and that it is likely a critical regulator of growth and developmental aspects of epithelial cells of the ovaries of laying hens as they transition from normal to a cancerous state. These results also provide a roadmap for our future research to investigate the precise role(s) of A2M in underlying mechanisms responsible for estrogen-mediated development of the chicken oviduct and as a biomarker to assess the effectiveness of therapies for endometrioid-type ovarian cancer in laying hens.

## Competing interests

The authors declare that they have no competing interests.

## Authors' contributions

WL performed all the experiments. WJ, JK, JL and JK contributed to the tissue sampling and the experiments. FWB participated in data analysis and drafted the manuscript. JH participated in the design of the study and providing all animals for tissue sampling. GS, as a corresponding author, designed the experiments, analyzed experimental data and drafted the manuscript. All authors read and approved the final manuscript.

## Supplementary Material

Additional file 1 **Supplemental Figure S1**. Multiple sequence alignment of chicken, mammalian and amphibian A2M proteins. (A) The protein sequences of A2M from chicken (*Gallus gallus)*, human (*Homo sapiens)*, chimpanzee (*Pan troglodytes)*, orangutan (*Pongo abelii*), rhesus monkey (*Macaca mulatta*), mouse (*Mus musculus)*, rat (*Rattus norvegicus)*, cow (*Bos taurus*) and frog (*Xenopus laevis*) were aligned using Geneious Pro Version 5.04 [39] with default penalties for gap and the protein weight matrix of BLOSUM (Blocks Substitution Matrix). Chicken A2M protein has moderate homology to mammalian A2M proteins (42.4-45.4%) and contains the highly conserved MG1 and MG2 domains, the A2M family N-terminal region, and the A2M receptor binding domain found in mammalian A2M. Shaded sequences indicate identical amino acid sequences among all species examined. Dashes represent gaps among the sequences. The conserved functional domains in A2M proteins were identified using the Pfam-A family matrix and NCBI conserved domain database. Click here for file

Additional file 2 **Supplemental Figure S2**. The phylogenetic tree generated from alignments of primary sequences of chicken, mammalian and amphibian A2M proteins. Results of phylogenetic tree analysis indicated that chicken A2M was between mammalian and amphibian species consistent with the general pattern of molecular evolution in vertebrates. Bootstrap values from 1,000 replicates are shown at the appropriate branches. Click here for file

Additional file 3 **Supplemental Figure S3**. Histological types of ovarian cancers in chickens used in this study. Briefly, clear cell carcinoma showed vacuolated cells consisting of nuclear atypia. Serous carcinoma was a solid mass of cells with nuclear atypia. Endometrioid carcinoma had many glands and mucinous carcinoma was differentiated around the stromal region. Each image shows two different regions within each type of carcinoma. Legend: Lane 1, clear cell carcinoma; Lane 2, serous/endometrioid/mucinous carcinoma; Lane 3, endometrioid carcinoma; Lane 4, serous carcinoma; Lane 5, endometrioid/mucinous carcinoma; Lane 6, endometrioid carcinoma; Lane 7, endometrioid carcinoma; Lane 8, clear cell carcinoma; Lane 9, serous/mucinous carcinoma; and Lane 10, serous/endometrioid/mucinous carcinoma. Click here for file

## References

[B1] Van Leuven F Marynen P Cassiman JJ Van den Berghe H Proteolysis of human alpha 2-macroglobulin without hydrolysis of the internal thiolesters or expression of the receptor recognition site J Biol Chem 1988 263 1 468 471 2447076

[B2] Sottrup-Jensen L Alpha-macroglobulins: structure, shape, and mechanism of proteinase complex formation J Biol Chem 1989 264 20 11539 11542 2473064

[B3] Armstrong PB Proteases and protease inhibitors: a balance of activities in host-pathogen interaction Immunobiology 2006 211 4 263 281 10.1016/j.imbio.2006.01.002 16697919

[B4] Tayade C Esadeg S Fang Y Croy BA Functions of alpha 2 macroglobulins in pregnancy Mol Cell Endocrinol 2005 245 (1 2):60-66 10.1016/j.mce.2005.09.011 16297527

[B5] Matthijs G Devriendt K Cassiman JJ Van den Berghe H Marynen P Structure of the human alpha-2 macroglobulin gene and its promotor Biochem Biophys Res Commun 1992 184 2 596 603 10.1016/0006-291X(92)90631-T 1374237

[B6] Sottrup-Jensen L Stepanik TM Kristensen T Wierzbicki DM Jones CM Lonblad PB Magnusson S Petersen TE Primary structure of human alpha 2-macroglobulin. V. The complete structure J Biol Chem 1984 259 13 8318 8327 6203908

[B7] Blacker D Wilcox MA Laird NM Rodes L Horvath SM Go RC Perry R Watson B Bassett SS McInnis MG Alpha-2 macroglobulin is genetically associated with Alzheimer disease Nat Genet 1998 19 4 357 360 10.1038/1243 9697696

[B8] Umans L Serneels L Overbergh L Lorent K Van Leuven F Van den Berghe H Targeted inactivation of the mouse alpha 2-macroglobulin gene J Biol Chem 1995 270 34 19778 19785 10.1074/jbc.270.34.19778 7544347

[B9] Zbroja-Sontag W Defense proteins and immune complexes in the blood serum of women with inflammatory and neoplastic lesions of the ovary Am J Reprod Immunol 1983 4 1 11 20 6194700 10.1111/j.1600-0897.1983.tb00247.x

[B10] Vaughan JM Vale WW Alpha 2-macroglobulin is a binding protein of inhibin and activin Endocrinology 1993 132 5 2038 2050 10.1210/en.132.5.2038 7682939

[B11] McElhinney B Ardill J Caldwell C Lloyd F McClure N Ovarian hyperstimulation syndrome and assisted reproductive technologies: why some and not others? Hum Reprod 2002 17 6 1548 1553 10.1093/humrep/17.6.1548 12042276

[B12] Yamada Y Aketa K Ovostatin, an endogenous trypsin inhibitor of sea urchin eggs: purification and characterization of ovostatin from eggs of the sea urchin, Strongylocentrotus intermedius Gamete Res 1988 19 3 265 275 10.1002/mrd.1120190306 3058564

[B13] Nagase H Harris ED Woessner JF Brew K Ovostatin: a novel proteinase inhibitor from chicken egg white. I. Purification, physicochemical properties, and tissue distribution of ovostatin J Biol Chem 1983 258 12 7481 7489 6408074

[B14] Nagase H Harris ED Jr Ovostatin: a novel proteinase inhibitor from chicken egg white. II. Mechanism of inhibition studied with collagenase and thermolysin J Biol Chem 1983 258 12 7490 7498 6305943

[B15] Nagase H Brew K Harris ED Jr Ovostatin: a proteinase inhibitor in egg whites that is homologous to alpha 2-macroglobulin Prog Clin Biol Res 1985 180 283 285 2412236

[B16] Dougherty DC Sanders MM Estrogen action: revitalization of the chick oviduct model Trends Endocrinol Metab 2005 16 9 414 419 10.1016/j.tem.2005.09.001 16202618

[B17] Hewitt SC Harrell JC Korach KS Lessons in estrogen biology from knockout and transgenic animals Annu Rev Physiol 2005 67 285 308 10.1146/annurev.physiol.67.040403.115914 15709960

[B18] Louet JF LeMay C Mauvais-Jarvis F Antidiabetic actions of estrogen: insight from human and genetic mouse models Curr Atheroscler Rep 2004 6 3 180 185 10.1007/s11883-004-0030-9 15068742

[B19] Pearce ST Jordan VC The biological role of estrogen receptors alpha and beta in cancer Crit Rev Oncol Hematol 2004 50 1 3 22 10.1016/j.critrevonc.2003.09.003 15094156

[B20] Wise PM Dubal DB Rau SW Brown CM Suzuki S Are estrogens protective or risk factors in brain injury and neurodegeneration? Reevaluation after the Women's health initiative Endocr Rev 2005 26 3 308 312 10.1210/er.2004-0014 15851820

[B21] Herynk MH Fuqua SA Estrogen receptor mutations in human disease Endocr Rev 2004 25 6 869 898 10.1210/er.2003-0010 15583021

[B22] Socher SH Omalley BW Estrogen-Mediated Cell-Proliferation during Chick Oviduct Development and Its Modulation by Progesterone Dev Biol 1973 30 2 411 417 10.1016/0012-1606(73)90098-5

[B23] Palmiter RD Wrenn JT Interaction of Estrogen and Progesterone in Chick Oviduct Development .3. Tubular Gland Cell Cytodifferentiation J Cell Biol 1971 50 3 598 & 10.1083/jcb.50.3.598 4329151 PMC2108294

[B24] Oka T Schimke RT Interaction of estrogen and progesterone in chick oviduct development. II. Effects of estrogen and progesterone on tubular gland cell function J Cell Biol 1969 43 1 123 137 10.1083/jcb.43.1.123 5350180 PMC2107849

[B25] Oka T Schimke RT Interaction of estrogen and progesterone in chick oviduct development. I. Antagonistic effect of progesterone on estrogen-induced proliferation and differentiation of tubular gland cells J Cell Biol 1969 41 3 816 831 10.1083/jcb.41.3.816 5814004 PMC2107830

[B26] Oka T Schimke RT Progesterone antagonism of estrogen-induced cytodifferentiation in chick oviduct Science 1969 163 862 83 85 10.1126/science.163.3862.83 5763496

[B27] Palmiter RD Wrenn JT Interaction of estrogen and progesterone in chick oviduct development. 3. Tubular gland cell cytodifferentiation J Cell Biol 1971 50 3 598 615 10.1083/jcb.50.3.598 4329151 PMC2108294

[B28] Bar A Differential Regulation of Calbindin in the Calcium-Transporting Organs of Birds with High Calcium Requirements J Poult Sci 2009 46 4 267 285 10.2141/jpsa.46.267

[B29] Hincke MT Nys Y Gautron J The Role of Matrix Proteins in Eggshell Formation J Poult Sci 2010 47 3 208 219 10.2141/jpsa.009122

[B30] Song G Seo HW Choi JW Rengaraj D Kim TM Lee BR Kim YM Yun TW Jeong JW Han JY Discovery of Candidate Genes and Pathways Regulating Oviduct Development in Chickens Biol Reprod 2011 85 306 314 10.1095/biolreprod.110.089227 21543768

[B31] Buhi WC Alvarez IM Kouba AJ Oviductal regulation of fertilization and early embryonic development J Reprod Fertil Suppl 1997 52 285 300 9602736

[B32] Okada A Sato T Ohta Y Iguchi T Sex steroid hormone receptors in the developing female reproductive tract of laboratory rodents J Toxicol Sci 2005 30 2 75 89 10.2131/jts.30.75 15928456

[B33] Lee SI Lee WK Shin JH Han BK Moon S Cho S Park T Kim H Han JY Sexually dimorphic gene expression in the chick brain before gonadal differentiation Poult Sci 2009 88 5 1003 1015 10.3382/ps.2008-00197 19359689

[B34] Sanders MM McKnight GS Positive and negative regulatory elements control the steroid-responsive ovalbumin promoter Biochemistry 1988 27 17 6550 6557 10.1021/bi00417a053 3064812

[B35] Seo HW Park JY Lee HC Kim D Song YS Lim JM Song G Han JY Physiological Effects of Diethylstilbestrol Exposure on the Development of the Chicken Oviduct J Anim Sci & Technol 2009 51 6 485 492 10.5187/JAST.2009.51.6.48521984724

[B36] Kohler PO Grimley PM O'Malley BW Estrogen-induced cytodifferentiation of the ovalbumin-secreting glands of the chick oviduct J Cell Biol 1969 40 1 8 27 10.1083/jcb.40.1.8 5782453 PMC2107600

[B37] McKnight GS The induction of ovalbumin and conalbumin mRNA by estrogen and progesterone in chick oviduct explant cultures Cell 1978 14 2 403 413 10.1016/0092-8674(78)90125-3 566622

[B38] Barua A Bitterman P Abramowicz JS Dirks AL Bahr JM Hales DB Bradaric MJ Edassery SL Rotmensch J Luborsky JL Histopathology of ovarian tumors in laying hens: a preclinical model of human ovarian cancer Int J Gynecol Cancer 2009 19 4 531 539 10.1111/IGC.0b013e3181a41613 19509547 PMC2759668

[B39] Drummond AJ Ashton B Buxton S Cheung M Cooper A Duran C Field M Heled J Kearse M Markowitz S Moir R Stones-Havas S Sturrock S Thierer T Wilson A Geneious v5.4 2011 http://www.geneious.com/

[B40] Song G Bazer FW Spencer TE Pregnancy and interferon tau regulate RSAD2 and IFIH1 expression in the ovine uterus Reproduction 2007 133 1 285 295 10.1530/REP-06-0092 17244754

[B41] Livak KJ Schmittgen TD Analysis of relative gene expression data using real-time quantitative PCR and the 2(-Delta Delta C(T)) Method Methods 2001 25 4 402 408 10.1006/meth.2001.1262 11846609

[B42] Fredrickson TN Ovarian tumors of the hen Environ Health Perspect 1987 73 35 51 3665870 10.1289/ehp.877335 PMC1474556

[B43] Munck Petersen C Christiansen BS Heickendorff L Ingerslev J Synthesis and secretion of alpha 2-macroglobulin by human hepatocytes in culture Eur J Clin Invest 1988 18 5 543 548 10.1111/j.1365-2362.1988.tb01054.x 2465899

[B44] de Sain-van der Velden MG Rabelink TJ Reijngoud DJ Gadellaa MM Voorbij HA Stellaard F Kaysen GA Plasma alpha 2 macroglobulin is increased in nephrotic patients as a result of increased synthesis alone Kidney Int 1998 54 2 530 535 10.1046/j.1523-1755.1998.00018.x 9690220

[B45] Anderson NL Anderson NG The human plasma proteome: history, character, and diagnostic prospects Mol Cell Proteomics 2002 1 11 845 867 10.1074/mcp.R200007-MCP200 12488461

[B46] Schaller J Gerber S Kaempfer U Trachsel C Human Blood Plasma Proteins, Structure and Function 2008 1 Wiley 845 867

[B47] Mann K The chicken egg white proteome Proteomics 2007 7 19 3558 3568 10.1002/pmic.200700397 17722208

[B48] Saxena I Tayyab S Protein proteinase inhibitors from avian egg whites Cell Mol Life Sci 1997 53 1 13 23 10.1007/PL00000575 9117993 PMC11147361

[B49] Kohler PO Grimley PM O'Malley BW Protein synthesis: differential stimulation of cell-specific proteins in epithelial cells of chick oviduct Science 1968 160 823 86 87 10.1126/science.160.3823.86 4868141

[B50] Jemal A Siegel R Ward E Murray T Xu J Thun MJ Cancer statistics, 2007 CA Cancer J Clin 2007 57 1 43 66 10.3322/canjclin.57.1.43 17237035

[B51] Wong AS Auersperg N Ovarian surface epithelium: family history and early events in ovarian cancer Reprod Biol Endocrinol 2003 1 70 10.1186/1477-7827-1-70 14609432 PMC270003

[B52] Cvetkovic D Early events in ovarian oncogenesis Reprod Biol Endocrinol 2003 1 68 10.1186/1477-7827-1-68 14577833 PMC239895

[B53] Goodman MT Correa CN Tung KH Roffers SD Cheng Wu X Young JL Wilkens LR Carney ME Howe HL Stage at diagnosis of ovarian cancer in the United States, 1992-1997 Cancer 2003 97 10 Suppl 2648 2659 12733130 10.1002/cncr.11347

[B54] Bast RC Urban N Shridhar V Smith D Zhang Z Skates S Lu K Liu J Fishman D Mills G Early detection of ovarian cancer: promise and reality Cancer Treat Res 2002 107 61 97 11775462 10.1007/978-1-4757-3587-1_3

[B55] Pepe MS Etzioni R Feng Z Potter JD Thompson ML Thornquist M Winget M Yasui Y Phases of biomarker development for early detection of cancer J Natl Cancer Inst 2001 93 14 1054 1061 10.1093/jnci/93.14.1054 11459866

[B56] Vanderhyden BC Shaw TJ Ethier JF Animal models of ovarian cancer Reprod Biol Endocrinol 2003 1 67 10.1186/1477-7827-1-67 14613552 PMC270002

[B57] Stakleff KD Von Gruenigen VE Rodent models for ovarian cancer research Int J Gynecol Cancer 2003 13 4 405 412 10.1046/j.1525-1438.2003.13317.x 12911715

[B58] Giles JR Shivaprasad HL Johnson PA Ovarian tumor expression of an oviductal protein in the hen: a model for human serous ovarian adenocarcinoma Gynecol Oncol 2004 95 3 530 533 10.1016/j.ygyno.2004.07.061 15581958

[B59] Ahn SE Choi JW Rengaraj D Seo HW Lim W Han JY Song G Increased expression of cysteine cathepsins in ovarian tissue from chickens with ovarian cancer Reprod Biol Endocrinol 2010 8 100 10.1186/1477-7827-8-100 20727192 PMC2931516

[B60] Lopez-Otin C Matrisian LM Emerging roles of proteases in tumour suppression Nat Rev Cancer 2007 7 10 800 808 10.1038/nrc2228 17851543

[B61] Koo PH Human alpha 2-macroglobulin: a major serum factor cytotoxic for tumor cells Cancer Lett 1983 18 2 169 177 10.1016/0304-3835(83)90064-2 6187436

[B62] Koo PH Tumor inhibition by human alpha 2-macroglobulin Ann N Y Acad Sci 1983 421 388 390 10.1111/j.1749-6632.1983.tb18129.x 6202208

[B63] Hawkridge AM Wysocky RB Petitte JN Anderson KE Mozdziak PE Fletcher OJ Horowitz JM Muddiman DC Measuring the intra-individual variability of the plasma proteome in the chicken model of spontaneous ovarian adenocarcinoma Anal Bioanal Chem 2010 398 2 737 749 10.1007/s00216-010-3979-y 20640409 PMC3140420

[B64] de Boer JP Creasey AA Chang A Abbink JJ Roem D Eerenberg AJ Hack CE Taylor FB Jr Alpha-2-macroglobulin functions as an inhibitor of fibrinolytic, clotting, and neutrophilic proteinases in sepsis: studies using a baboon model Infect Immun 1993 61 12 5035 5043 7693593 10.1128/iai.61.12.5035-5043.1993 PMC281280

