# Supplementary material for: Differential expression of alpha 2 macroglobulin in response to dietylstilbestrol and in ovarian carcinomas in chickens
Source: Reprod Biol Endocrinol. 2011 Oct 7;9:137. doi: 10.1186/1477-7827-9-137 (PMC3204285; doi:10.1186/1477-7827-9-137)
Supplement: Additional file 1 — Supplemental Figure S1. Multiple sequence alignment of chicken, mammalian and amphibian A2M proteins. (A) The protein sequences of A2M from chicken (Gallus gallus), human (Homo sapiens), chimpanzee (Pan troglodytes), orangutan (Pongo abelii), rhesus monkey (Macaca mulatta), mouse (Mus musculus), rat (Rattus norvegicus), cow (Bos taurus) and frog (Xenopus laevis) were aligned using Geneious Pro Version 5.04 [39] with default penalties for gap and the protein weight matrix of BLOSUM (Blocks Substitution Matrix). Chicken A2M protein has moderate homology to mammalian A2M proteins (42.4-45.4%) and contains the highly conserved MG1 and MG2 domains, the A2M family N-terminal region, and the A2M receptor binding domain found in mammalian A2M. Shaded sequences indicate identical amino acid sequences among all species examined. Dashes represent gaps among the sequences. The conserved functional domains in A2M proteins were identified using the Pfam-A family matrix and NCBI conserved domain database. [file 1477-7827-9-137-S1.PDF]

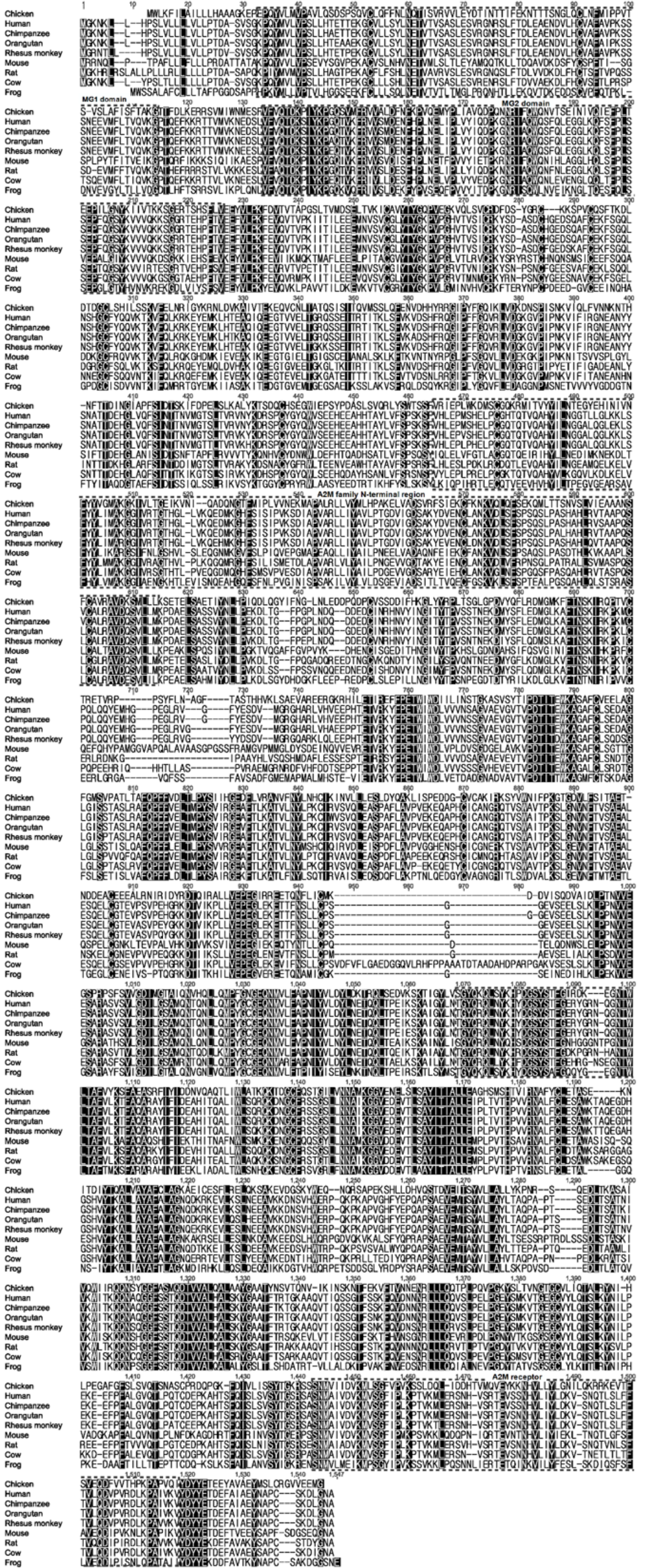
Chicken
Human
Chimpanzee
Orangutan
Rhesus monkey
Mouse
Rat
Cow
Frog
MG1 domain
MG2 domain
A2M family N-terminal region
A2M receptor
