# Supplementary figures and images for: Differential expression of alpha 2 macroglobulin in response to dietylstilbestrol and in ovarian carcinomas in chickens
Source: Reprod Biol Endocrinol. 2011 Oct 7;9:137. doi: 10.1186/1477-7827-9-137 (PMC3204285; doi:10.1186/1477-7827-9-137)

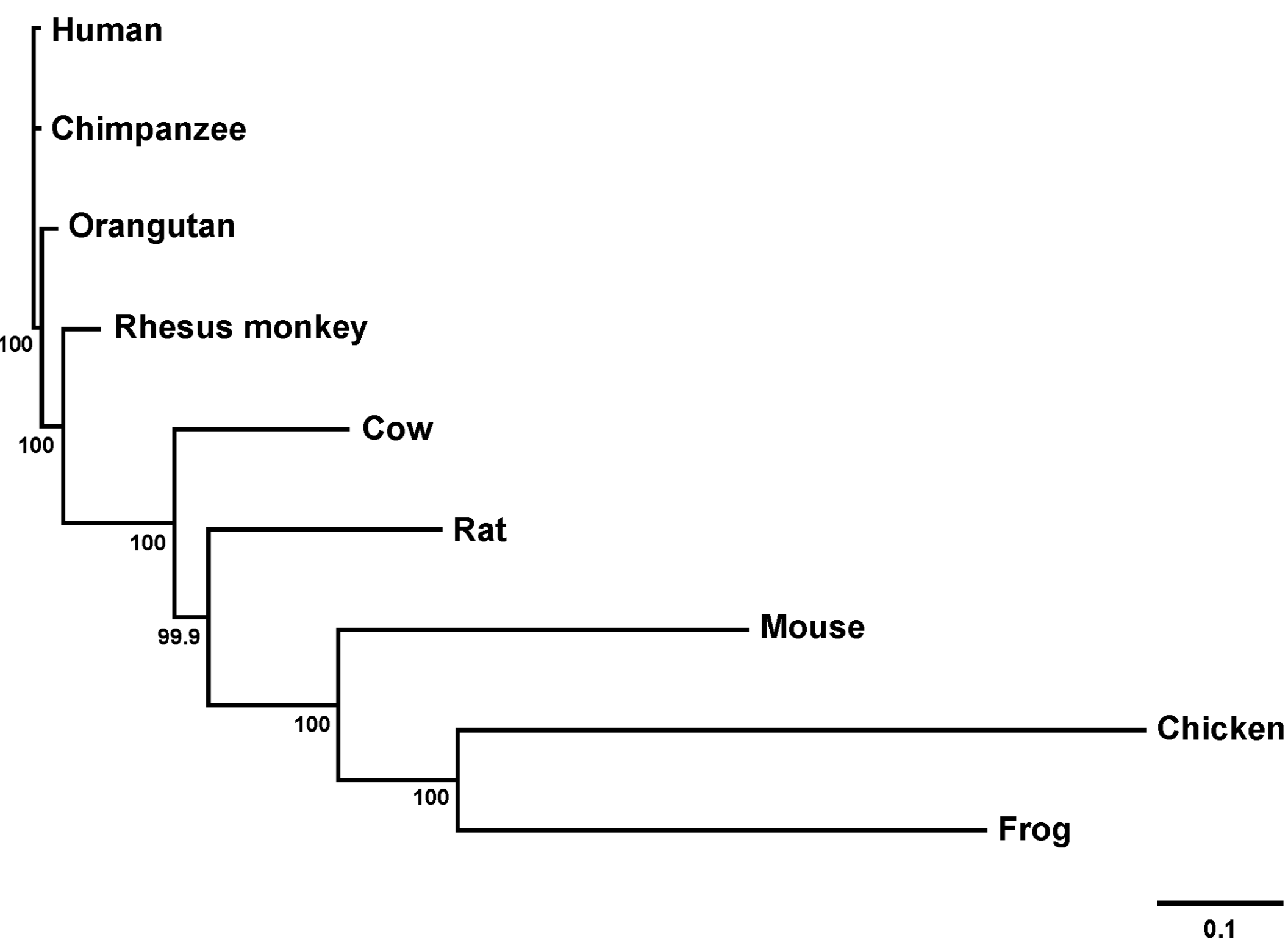

Supplement: Additional file 2 — Supplemental Figure S2. The phylogenetic tree generated from alignments of primary sequences of chicken, mammalian and amphibian A2M proteins. Results of phylogenetic tree analysis indicated that chicken A2M was between mammalian and amphibian species consistent with the general pattern of molecular evolution in vertebrates. Bootstrap values from 1,000 replicates are shown at the appropriate branches. [file 1477-7827-9-137-S2.PDF]

1

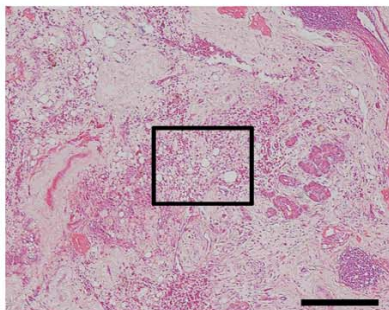

2

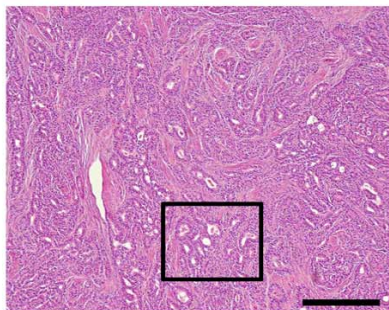

3

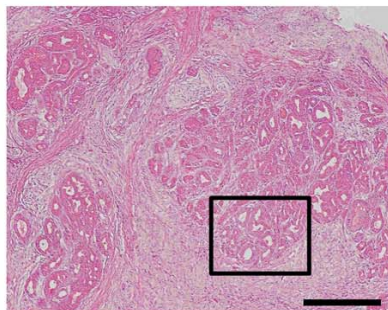

4

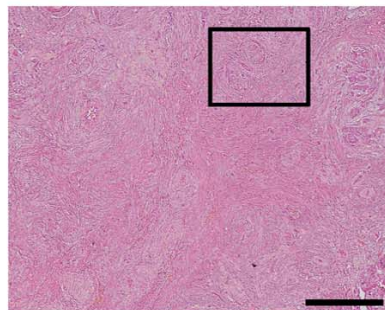

5

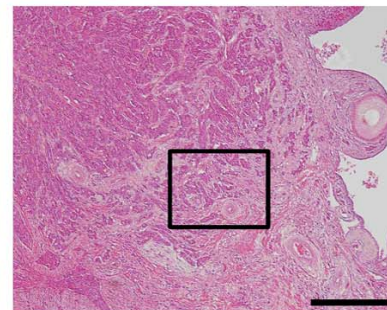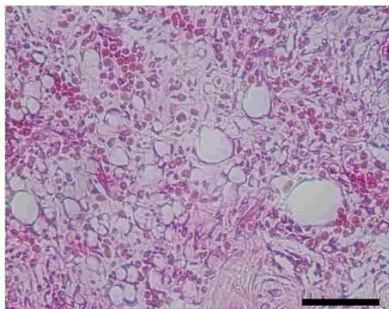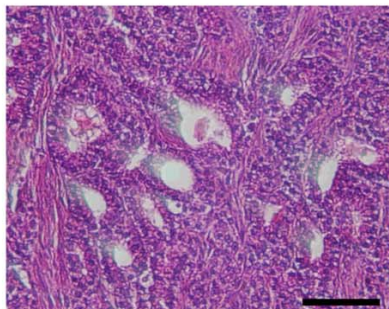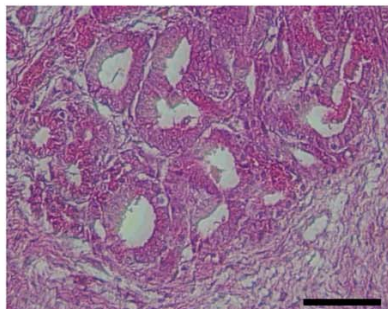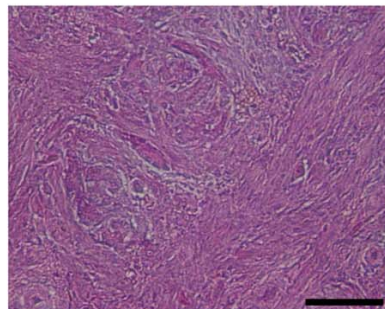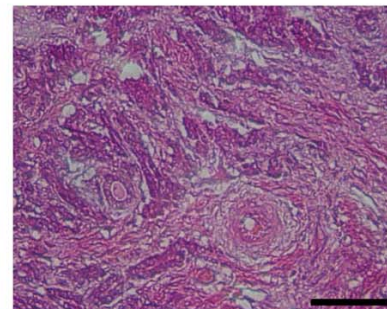

6

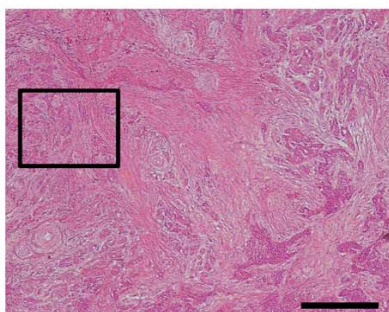

7

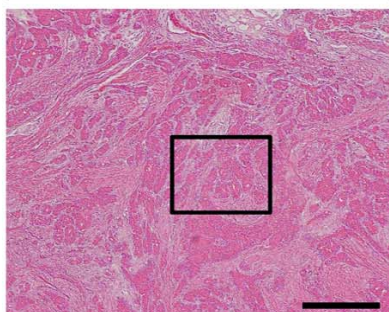

8

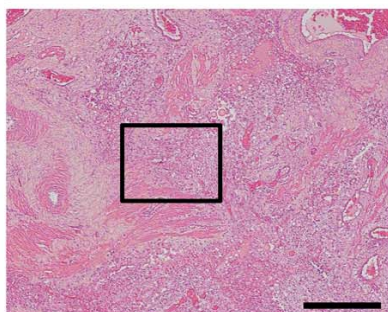

9

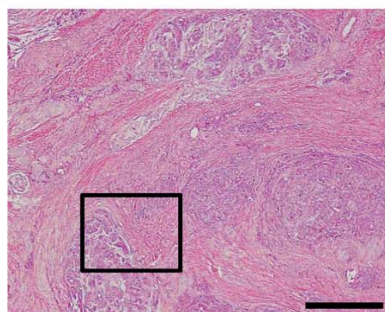

10

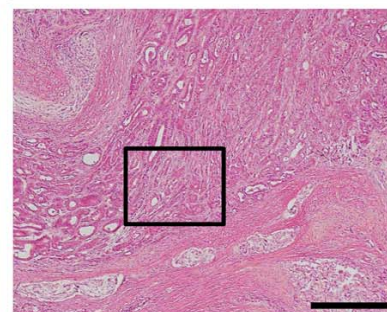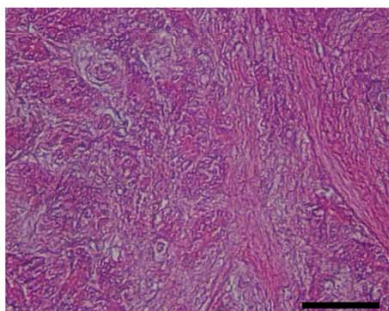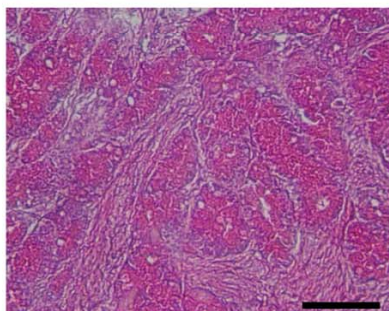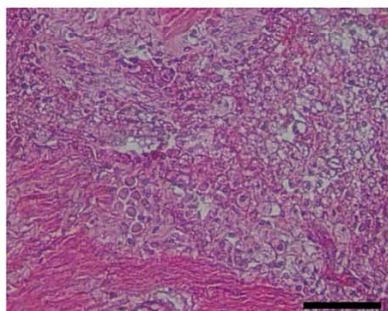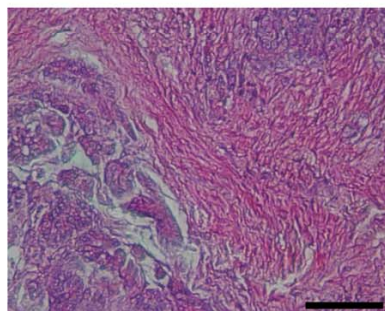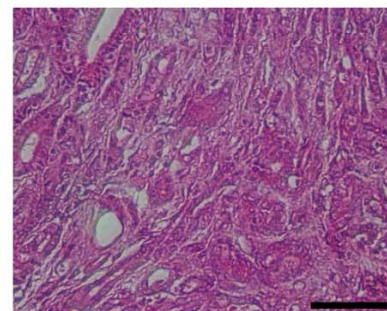

Supplement: Additional file 3 — Supplemental Figure S3. Histological types of ovarian cancers in chickens used in this study. Briefly, clear cell carcinoma showed vacuolated cells consisting of nuclear atypia. Serous carcinoma was a solid mass of cells with nuclear atypia. Endometrioid carcinoma had many glands and mucinous carcinoma was differentiated around the stromal region. Each image shows two different regions within each type of carcinoma. Legend: Lane 1, clear cell carcinoma; Lane 2, serous/endometrioid/mucinous carcinoma; Lane 3, endometrioid carcinoma; Lane 4, serous carcinoma; Lane 5, endometrioid/mucinous carcinoma; Lane 6, endometrioid carcinoma; Lane 7, endometrioid carcinoma; Lane 8, clear cell carcinoma; Lane 9, serous/mucinous carcinoma; and Lane 10, serous/endometrioid/mucinous carcinoma. [file 1477-7827-9-137-S3.PDF]
